# Artificial intelligence approaches for anti-addiction drug discovery

**DOI:** 10.1039/d5dd00032g

**Published:** 2025-05-13

**Authors:** Dong Chen, Jian Jiang, Nicole Hayes, Zhe Su, Guo-Wei Wei

**Affiliations:** a Department of Mathematics, Michigan State University MI 48824 USA weig@msu.edu; b Research Center of Nonlinear Science, School of Mathematical and Physical Sciences, Wuhan Textile University Wuhan 430200 P. R. China; c Department of Electrical and Computer Engineering, Michigan State University MI 48824 USA; d Department of Biochemistry and Molecular Biology, Michigan State University MI 48824 USA

## Abstract

Drug addiction remains a complex global public health challenge, with traditional anti-addiction drug discovery hindered by limited efficacy and slow progress in targeting intricate neurochemical systems. Advanced algorithms within artificial intelligence (AI) present a transformative solution that boosts both speed and precision in therapeutic development. This review examines how artificial intelligence serves as a crucial element in developing anti-addiction medications by targeting the opioid system along with dopaminergic and GABAergic systems, which are essential in addiction pathology. It identifies upcoming trends promising in studying less-researched addiction-linked systems through innovative general-purpose drug discovery techniques. AI holds the potential to transform anti-addiction research by breaking down conventional limitations, which will enable the development of superior treatment methods.

## Abstract 2

AI-driven drug discovery accelerates anti-addiction treatment by enhancing precision and targeting key neurochemical systems.

## Graphical abstract

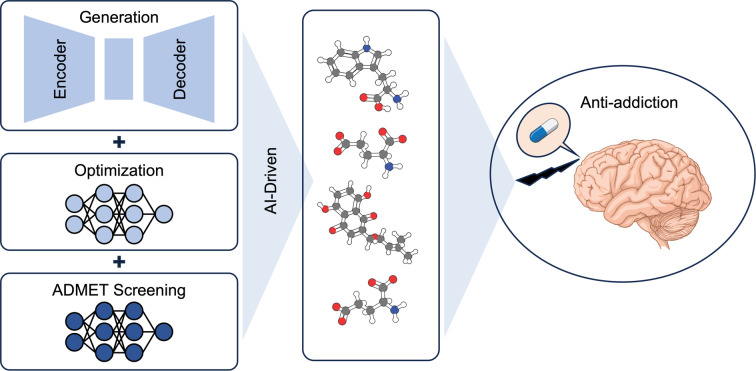

## 1 Introduction

Drug addiction is a chronic relapsing disease that affects millions of people globally and poses significant social, economic, and health challenges.^1–4^ In the United States alone, over 48 million people aged 12 and older battled a substance use disorder in 2023, yet fewer than 10% received treatment, underscoring a critical gap in care.^5^ Globally, the World Health Organization estimates that 35 million individuals suffer from drug use disorders, with overdose deaths—particularly from opioids—surging to over 107 000 in the U.S. in 2023.^6^ Beyond these alarming statistics, addiction fractures families, drives economic costs exceeding 600 billion dollars annually in the U.S. through healthcare expenses, lost productivity, and criminal justice involvement, and perpetuates a cycle of social and health inequities. This pervasive crisis demands urgent attention because traditional approaches to treatment and drug discovery have struggled to keep pace with its scale and complexity, leaving a pressing need for innovative solutions.

The current public health emergency caused by addiction demands immediate intervention efforts. The opioid epidemic continues to pose major challenges by 2025 as synthetic opioids like fentanyl remain widely accessible. Treatment efforts become more challenging due to the rising occurrence of polydrug use and substance use disorders involving methamphetamine and alcohol alongside other drugs.^7^ Despite improved public awareness from media and advocacy efforts, the stigma around addiction continues because only one in four Americans view it as a medical issue instead of a moral failing.^8^ The SUPPORT Act's implementation of increased harm reduction funding and medication-assisted treatment funding in the United States indicates significant policy advancement. Access to essential services continues to show significant gaps, particularly among underprivileged communities. However, disparities in access remain, particularly in underserved communities. The dynamic environment of substance use disorders, which shows high prevalence rates alongside limited treatment adoption and changing societal perceptions, demands immediate progress in addiction therapeutics to meet clinical needs and reduce the global burden of these disorders.

The global issue of addiction treatment demands new approaches to improve intervention effectiveness. The development of anti-addiction drugs serves as a crucial element in reducing the harmful impact of addiction. The primary goal of current research in addiction therapy has been to create therapeutic drugs that change addictive behaviors, diminish withdrawal symptoms, and prevent relapse.^9^ However, the pathway to developing drugs for addiction treatment encounters numerous challenges, such as the complex neurobiology of substance use disorders (SUDs), the high drop-out rates in clinical trials, and the extended time frames typical of conventional methods. Though empirical screening and receptor-based drug design have produced significant therapeutic insights and treatments,^10^ these methods face limitations when attempting to address addiction's complex mechanisms.^11–13^ Artificial intelligence (AI) functions as a transformative tool by applying advanced algorithms to reshape drug discovery processes. AI boosts process efficiency and accuracy through data analysis while providing solutions to overcome conventional obstacles.

This review examines AI-driven methods in developing anti-addiction drugs and demonstrates AI's transformative impact on addressing historical challenges in addiction research.^9,14–17^ The scope of this review encompasses both the biological mechanisms of addiction and their associated molecular targets. It also explores methods to reduce addiction and shows how AI utilizes advanced predictive methods from target identification to compound optimization to speed up the creation of effective treatments and predict addiction risks.^18–21^ Finally, this review looks to future possibilities by fine-tuning the general-purpose models and exploring new AI-led approaches that show potential to improve addiction prevention and treatment methods. This review takes a structured approach to understand how AI-based models drive innovation in anti-addiction drug discovery while demonstrating their ability to resolve existing challenges and push forward effective therapeutic development.

## 2 Biological mechanisms and related targets of addiction

### 2.1 Biological mechanisms of addiction

Addiction functions as a chronic condition that involves repeated relapses and compulsive drug-seeking behavior even when users face harmful effects. The brain's reward system and motivation circuits undergo maladaptive changes alongside its inhibitory control mechanisms, which result in substance use disorder (SUD). SUD encompasses a broader spectrum of problematic substance use, ranging from mild to severe. The biological basis of addiction involves the dysregulation of several key neurochemical pathways. The mesolimbic pathway releases more dopamine, which strengthens drug-seeking behavior through the dopaminergic reward system.^18^ The system responsible for synaptic plasticity and learning becomes altered to foster compulsive drug use according to research findings.^19^ Additionally, the γ-aminobutyric acid (GABA) system dysfunction reduces inhibitory control, which increases the difficulty of resisting cravings.^20,22^ Progression and severity of addiction development are influenced by additional pathways such as opioid, serotonergic, cannabinoid, and stress-related systems. A complex neurobiological framework that sustains addiction emerges from the interaction between systems influenced by genetic, epigenetic, and environmental factors.^23^ The intricate nature of addiction demands precise targeting of specific molecular pathways to develop successful treatments for SUD and its severe addiction-related manifestations.

### 2.2 Key molecular targets

The study of SUD occurs because of disruptions across several neurochemical pathways. The primary systems for addiction treatment focus on dopaminergic, opioid, glutamatergic, and GABAergic pathways, which have received FDA approval. The serotonergic pathway, alongside cannabinoid and stress-related pathways, affects both drug-seeking behavior and mood regulation, which makes them potential targets for experimental therapies. Developing effective treatments for SUD requires a clear understanding of the interactions between these neurochemical systems, which is especially important for treating severe addiction cases.

The dopamine transporter (DAT) and the dopamine receptors (D1–D5) within the mesolimbic dopamine pathway play essential roles in addiction by encouraging drug-seeking behaviors through reinforcement. Both amphetamines and cocaine work by inhibiting dopamine reuptake, which leads to prolonged dopamine activity in synapses, explaining their addictive nature.^24^ While no FDA-approved drugs currently target DAT for addiction treatment, the FDA has authorized bupropion, a norepinephrine–dopamine reuptake inhibitor (NDRI), for nicotine dependence, and scientists are studying its possible uses for stimulant use disorder.^25^

Opioid addiction is mediated by the μ-opioid receptor (mOR), which binds substances like heroin and fentanyl, producing analgesia and euphoria.^26^ FDA-approved treatments such as methadone (a full mOR agonist) and buprenorphine (a partial agonist) mitigate withdrawal symptoms and cravings, while naltrexone, an opioid receptor antagonist, blocks the effects of opioids and prevents relapse.^27^ In addition, the κ-opioid receptor (KOR) is a target,^28^ as its modulation may reduce stress-induced relapse, offering a potential avenue for future drug development.

The neurotransmitter glutamate plays an essential role in synaptic plasticity and learning processes that form memories and contributes to neuroadaptive changes related to addiction.^24^ The persistent use of drugs alters glutamate balance and triggers compulsive behaviors to seek drugs. The *N*-methyl-d-aspartate receptor (NMDAR) represents a promising target because the FDA-approved drug acamprosate modulates it to treat alcohol use disorder.^29^ Research has demonstrated that the metabotropic glutamate receptors mGluR2/3 could reduce drug cravings since experimental compounds targeting these receptors show promise in preclinical studies.^30^

The GABA system maintains critical inhibitory control functions since GABA-A receptors serve as the main action point for both alcohol and benzodiazepines.^31^ Alcohol increases GABA inhibition while long-term consumption results in neuroadaptations that create withdrawal symptoms. Research indicates that Baclofen, which activates GABA-B receptors might lessen substance cravings and withdrawal symptoms for alcohol and cocaine dependency cases. The FDA has not yet approved this treatment for SUD, but studies show it holds significant promise.^32^

The 5-HT2A and 5-HT2C receptors within the serotonergic system influence mood regulation and impulse control which explains their significance in addiction treatment.^33^ Selective serotonin reuptake inhibitors (SSRIs) have been investigated for treating patients with both depression and addiction but show limited effectiveness as direct treatments for substance use disorders.^34^ The cannabinoid receptor 1 (CB1) has gained research interest as a therapeutic target because of its significant influence on reward and stress-related responses. Rimonabant was a CB1 antagonist that appeared promising yet was taken off the market because of its psychiatric side effects.^35^

While the dopaminergic, opioid, glutamatergic, and GABA systems remain the primary focus of FDA-approved addiction treatments, serotonergic, cannabinoid, and stress-related pathways offer promising avenues for novel therapeutics. Ongoing research aims to refine these targets, addressing addiction through a combination of pharmacological and behavioral interventions.

## 3 AI-driven anti-addiction drug discovery

Traditional behavioral and psychosocial treatments have failed to prevent addiction's emergence and advancement because of the enduring biological drivers discussed in Section 2. The development of new anti-addiction medications serves as a fundamental solution through their ability to interrupt the molecular and chemical processes that drive addictive behavior. These medications work to block the rewarding effects, such as dopamine reuptake inhibitors for psychostimulants, while also alleviating withdrawal symptoms through opioid receptor modulators and reducing relapse risk with glutamate receptor antagonists, which provides a proactive strategy to prevent addiction.^9^ Traditional drug discovery processes have failed to produce effective solutions for addiction due to high failure rates among potential treatments, extended development periods and a failure to address the full scope of addiction complexity, which is reflected by the small number of FDA-approved medications for cocaine addiction.^14^ This gap underscores the urgent need for innovative approaches to anti-addiction drug discovery, where AI steps in as a transformative force in this area. AI-driven drug discovery enhances the effectiveness and accuracy of developing essential therapeutics, which presents a promising route to mitigate addiction before we explore data preparation and specialized methods in this section.

### 3.1 Datasets

AI applications perform best when they use high-quality data that has been properly integrated. AI models require robust datasets to train effectively because addiction involves complex biological mechanisms, including neurochemical pathways alongside genetic and environmental factors. Successful data preparation requires careful curation as well as processing and harmonization of various biological and molecular data sets. The establishment of a foundational step provides essential support to AI-driven solutions, which enables the identification of effective anti-addiction treatments.

Drug discovery relies heavily on molecular datasets to identify candidate molecules because they provide essential chemical and pharmacological information. General-purpose experimental databases like ChEMBL,^36^ PubChem,^37^ and DrugBank^38^ supply comprehensive datasets about drug-like molecules, which encompass their structural information, chemical properties, and biological target interactions. Researchers use these databases to construct predictive models and evaluate drug candidates while also searching for new applications of current treatments. DrugBank stands out in anti-addiction drug discovery because it brings together information about FDA-approved drugs, experimental therapeutics, and pharmacological characteristics.^38^

For addiction-focused studies, specialized molecular datasets provide deeper insight into receptor–ligand interactions and toxicity profiles. BindingDB, for instance, offers protein–ligand binding affinity data critical for understanding receptor–ligand dynamics in addiction-related pathways.^39^ To address safety considerations, toxicity-focused datasets such as Tox21 provide chemical toxicity information, aiding researchers in designing safer therapeutic agents.^40^ Complementing these experimental resources are computational datasets such as the ZINC database,^41^ which contains millions of virtual molecules suitable for virtual screening, allowing efficient exploration of chemical spaces and cost-effective identification of potential therapeutic compounds. Advancements in machine learning have further enhanced the utility of molecular datasets in drug discovery. For example, the MolData dataset compiles extensive PubChem drug screening results, facilitating molecular machine learning applications aimed at improving drug discovery processes.^42^ By providing a structured compilation of bioassay data, MolData enables the development of predictive models that can identify potential therapeutic compounds and repurposing opportunities across various diseases, including addiction.

Targeted molecular datasets are essential for addressing addiction-specific mechanisms and safety challenges. The main treatment method for psychostimulant drugs like cocaine targets dopamine reuptake inhibition through modulation of the DAT. The DAT dataset provides vital information about DAT interactions, which serve as a fundamental component in understanding addiction-related pathways.^43^ The provided dataset reveals the biochemical processes behind dopamine reuptake, which enables the development of compounds that control DAT function to directly target addiction itself. Developing DAT inhibitors requires thorough assessment of off-target interactions specifically with the human Ether-à-go-go-Related Gene (hERG) potassium channel, since blockade can trigger dangerous ventricular arrhythmias. The hERG dataset resolves this essential safety issue through evaluation of the cardiotoxic potential of candidate molecules.^43,44^ Researchers can use the combined DAT and hERG datasets to create addiction treatments that optimize both effectiveness and safety through precise targeting and stringent safety evaluation.^45,46^

The vital resources for drug target discovery and addiction mechanism comprehension include gene expression datasets like Gene Expression Omnibus (GEO),^47^ encyclopedia of DNA elements (ENCODE),^48^ and ArrayExpress.^49^ These datasets serve as critical resources specifically for addiction research and the creation of new anti-addiction medications. GEO offers a carefully curated collection of gene expression profiles that reveal patterns of gene and pathway imbalances during addiction. ENCODE offers a detailed catalog of the human genome's functional elements that contain transcriptional and epigenetic information to help researchers understand addiction-related gene regulatory systems and identify potential target genes. ArrayExpress archives functional genomics high-throughput experiment data, which researchers can use to study transcript alterations related to addiction.

Protein–protein interaction (PPI) databases such as STRING,^50^ BioGRID,^51^ and IntAct^52^ have also enriched the tools available in the drug discovery process by providing a framework for studying addiction at a systems level. Among them, the STRING dataset integrates experimental data and predicted data to map interaction networks, allowing researchers to identify proteins involved in addiction-related signaling pathways, such as proteins in dopaminergic and serotonergic systems found in Mering *et al.*'s^50^ studies. BioGRID provides experimentally validated data to discover high-confidence targets, while IntAct provides curated interaction profiles that can be used to prioritize addiction-related proteins.^51^ Together, these PPI datasets support pathway enrichment analysis and the identification of key proteins in addiction-related networks.

Complementing these datasets, the Human Protein Atlas^53^ links protein expression and localization data to specific tissues, including regions of the brain affected by addiction. This enables the identification of tissue-specific drug targets, minimizing off-target effects and improving treatment precision. The Comparative Toxicogenomics Database (CTD) links genetic data to chemical exposures, revealing how environmental agents affect addiction pathways and guiding therapeutic development.^54^ The Human Reference Interactome (HuRI) also provides a high-quality PPI network, highlighting central proteins (hubs) as major drug targets.^55^

By integrating these datasets, researchers can prioritize targets, perform pathway enrichment analysis, and conduct research and development for anti-addiction drugs. In addition, a collection of the molecular and biological datasets and their references is provided in Table 1.

**Table 1:** Summary of molecular and biological datasets

Molecular datasets	Biological datasets
PubChem^37^	STRING^50^
ChEMBL^36^	BioGRID^51^
DrugBank^38^	IntAct^52^
BindingDB^39^	Comparative toxicogenomics database (CTD)^54^
ZINC^41^	Human reference interactome (HuRI)^55^
MolData^42^	Gene expression omnibus (GEO)^47^
Tox21 (ref. 56)	Encyclopedia of DNA elements (ENCODE)^48^
GDB-17 (ref. 57)	ArrayExpress^49^
hERG^44^	Kyoto encyclopedia of genes and genomes (KEGG)^58^
DAT^45^	Protein data bank (PDB)^59^
	Open targets^60^

### 3.2 AI-based methods for anti-addiction drug discovery

AI methods specifically designed to overcome biological and therapeutic obstacles in addiction treatment build on earlier discussions related to anti-addiction therapeutic development. Anti-addiction research has adopted diverse machine learning methods over the past ten years which include gradient boosting decision tree (GBDT),^14,61^ artificial neural network (ANN),^14,62^ autoencoder,^14,63^ transformer,^43^ generative adversarial networks (GANs),^64^ large language models (LLMs),^65^ multitask deep neural networks (DNNs)^14,61^ and knowledge graphs.^66^ Researchers have developed advanced methodologies, including topology-inferred drug addiction learning (TIDAL)^43^ and multi-layer graph attention neural network (MLGANN).^62^ The majority of AI techniques for drug discovery are generally applicable in many contexts, but few specific methods have been developed to target pathways associated with addiction and optimize treatments for substance use disorders. This subsection examines the opioid system along with dopaminergic and GABAergic systems and explains how AI methods are speeding up the development of effective treatment options for these biological targets.

#### AI approaches to anti-addiction target discovery

**Fig. 1:**
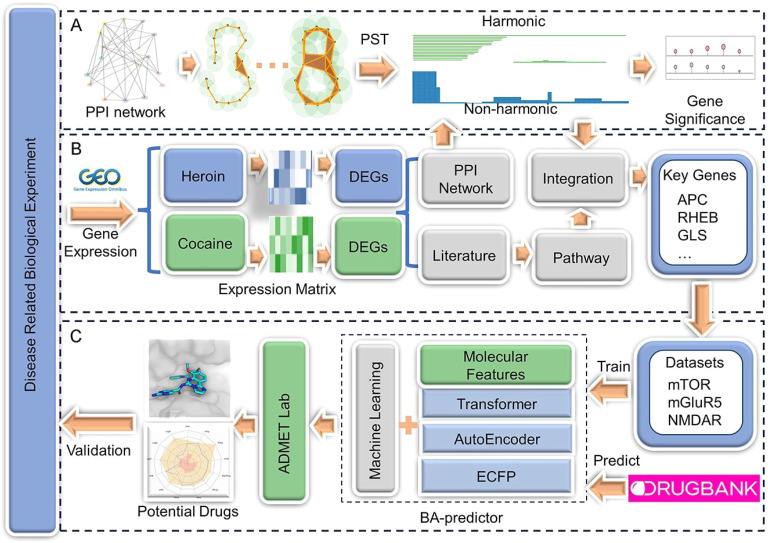
(A) PST-based topological differentiation analysis: the persistent spectral graph was used to identify key nodes in the PPI network. (B) Gene analysis: opioid- and cocaine-related transcriptomic data from GEO were analyzed to identify key genes through PPI networks, validated *via* literature and pathway analysis. (C) Drug repurposing: machine learning models predicted DrugBank compound affinities for addiction-related targets (mTOR, mGluR5, NMDAR), with ADMET analysis identifying potential treatments. Reproduced with permission from ref. 67. Copyright 2024 Oxford University Press.

As shown in Fig. 1, Du *et al.*^67^ employed topological data analysis (TDA) with persistent spectral theory (PST)^68^ to analyze protein–protein interaction (PPI) networks derived from opioid addiction transcriptomic data. Their pipeline started with differential gene expression (DEG) analysis, followed by PST-based topological differentiation to assess interaction strengths across multiple network thresholds.^69^ This approach identified mTOR, mGluR5, and NMDAR as key targets, validated through literature and pathway analysis, and subsequently integrated into ML models for drug repurposing. This work exemplifies a genomic-to-therapeutic workflow, enhancing target identification for opioid addiction.

#### AI approaches to the opioid system

The opioid system, pivotal in pain modulation and addiction, has been extensively studied using AI-driven approaches to identify targets and develop non-addictive treatments. Feng and Wei^70^ advanced this field by applying machine learning to explore opioid receptor networks, screening over 120 000 drug candidates to identify promising inhibitors targeting nociceptin receptors, a critical component in opioid use disorder (OUD). Their study also assessed ADMET (absorption, distribution, metabolism, excretion, and toxicity) properties, ensuring the safety and efficacy of potential compounds. Szymański *et al.*^71^ utilized AI-based virtual screening to evaluate billions of virtual molecules, discovering two compounds capable of relieving pain without the addictive properties of traditional opioids, such as those binding strongly to mORs.

**Fig. 2:**
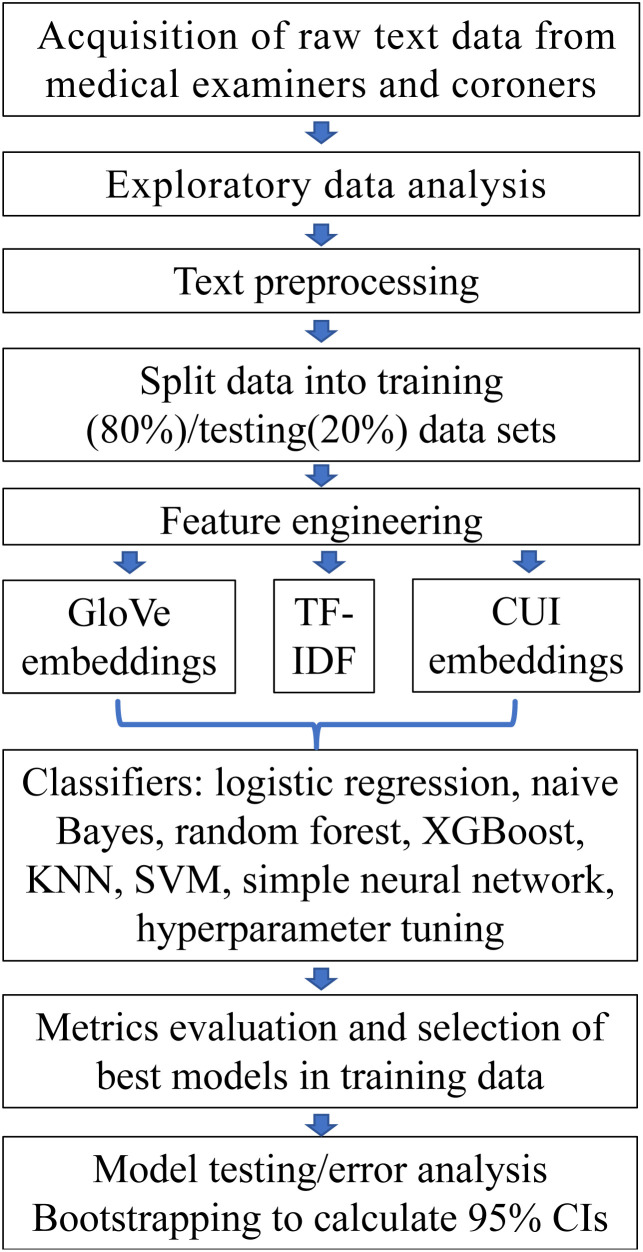
Natural language processing pipeline for substance classification in overdose fatalities. Reproduced with permission from ref. 73. Copyright 2022 American Medical Association.

Furthermore, more advanced AI-based approaches were introduced, such as Natural Language Processing (NLP) methods, which extract addiction-specific insights from unstructured biomedical literature, aiding target identification and hypothesis generation. Models such as those built on specific topics can help organize and group addiction research articles, making it easier to identify emerging trends and related research.^72^ This technology enables researchers to efficiently process large amounts of unstructured text and discover new connections that might otherwise be overlooked, which is particularly valuable for large-scale literature mining. For example, as shown in Fig. 2, Goodman-Meza *et al.*^73^ applied text mining techniques to classify substances associated with overdose deaths in 35 433 unstructured medical examination records in 2020 using NLP and ML. Using text mining methods, the study achieved excellent classification performance for substances such as opioids, methamphetamine, cocaine, and alcohol.

Additionally, drug repurposing research has found medications like tramadol, olanzapine, mirtazapine, bupropion, and atomoxetine as possible treatments for OUD.^67,74^ The development process becomes faster and cheaper because it utilizes existing safety profiles as well as pharmacokinetics and clinical data. Researchers can discover novel mechanisms of action and target shared neurobiological pathways between various substance use disorders through drug repurposing. The research conducted by Feng *et al.*^63^ and Angelo *et al.*^75^ advanced molecular design by implementing multi-objective optimization to target multiple opioid receptors while balancing efficacy with ADMET properties. A combination of AI techniques applied from target identification to lead optimization enables the development of successful non-addictive treatments for opioid addiction.

#### AI approaches to voltage-gated sodium channels

Since the opioid system mediates both analgesia and addiction, identifying alternative pain modulators could reduce opioid reliance, offering a strategy to mitigate addiction risk while addressing pain, a major driver of opioid misuse. Synthetic opioids such as fentanyl exhibit exceptionally strong binding to these receptors, and this mechanism has been extensively studied by Mahinthichaichan *et al.*^76^ Chen *et al.*^77^ explored pain management through a proteomic learning approach targeting voltage-gated sodium channels (Nav1.3, Nav1.7, Nav1.8, Nav1.9), which are critical for neuronal excitability in the peripheral nervous system. Using PPI and DTI networks, ML screens lead compounds to optimize efficacy and minimize side effects. Though focused on sodium channels, this work connects to opioid addiction through shared pain and reward pathways.

#### AI approaches to the dopaminergic system

**Fig. 3:**
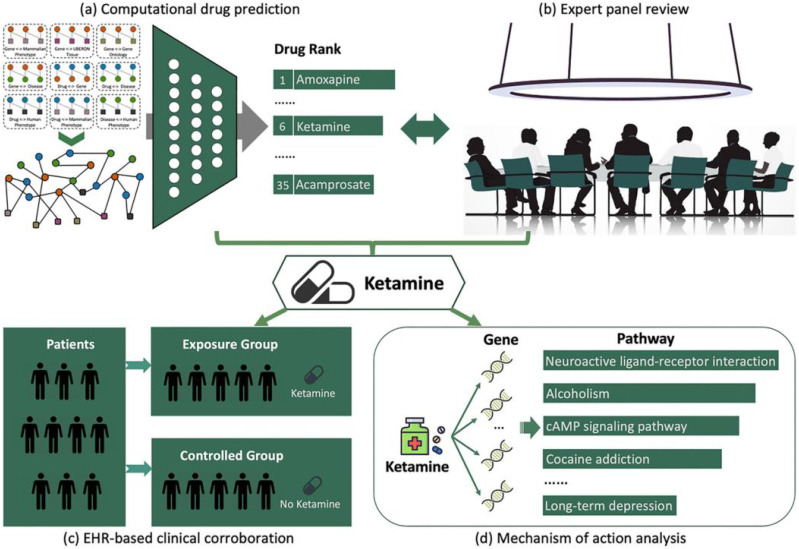
Overview of the drug repurposing pipeline for cocaine use disorder (CUD). (a) A knowledge-graph-based drug discovery system integrating multi-type interactions from diverse biomedical databases was utilized to rank potential candidate drugs for cocaine use disorder (CUD) treatment. (b) The CTN-0114 advisory committee evaluated the top-ranked candidates and selected ketamine for clinical investigation. (c) Insights from electronic health records provided clinical evidence supporting ketamine's potential effectiveness for CUD treatment. (d) Genetic and functional analyses revealed that ketamine directly interacts with multiple CUD-associated genes and pathways. Reproduced with permission from ref. 66. Copyright 2023 Wiley.

The dopaminergic system, central to the reward pathway and heavily implicated in addiction, has been a focus of AI-driven drug discovery efforts targeting key proteins like the DAT. Zhu *et al.*^43^ developed the topology-inferred drug addiction learning (TIDAL) framework, integrating multi-omics data (transcriptomics and proteomics) into a topology-inferred analysis of cocaine addiction PPI networks. This approach prioritizes DAT and other genes, offering a scalable workflow from data integration to target ranking, significantly enhancing target identification for cocaine addiction, which shares mechanisms with other addictions. Gao *et al.*^66^ utilized knowledge graphs (KGs) to predict drug–target interactions, identifying ketamine as a repurposed drug for cocaine use disorder by mapping relationships involving DAT and serotonin transporters (SERT). Their integrated strategy combined KG-based predictions, expert review, clinical data, and mechanism analysis, providing a network-driven perspective on addiction pathways. This approach also highlights key targets like dopamine and serotonin transporters (DAT, SERT), offering a network-driven perspective on addiction pathways. Such KG methodologies enhance target identification and drug repurposing, directly addressing the scarcity of addiction-specific treatments. In addition, advanced models such as multi-layer graph attention neural network (MLGANN)^62^ improve drug–target interaction predictions by combining multi-source data and network heterogeneity, offering unparalleled precision. Using these knowledge-graph-based approaches, researchers can accelerate the discovery of new targets and potential treatments for addiction, ultimately improving the drug development process in this critical area of research (Fig. 3).

**Fig. 4:**
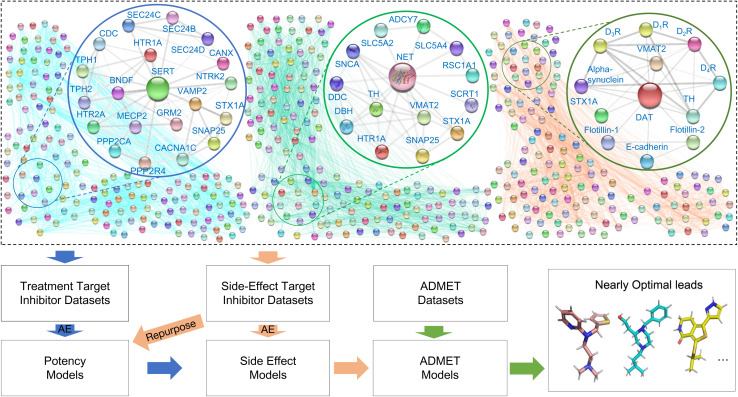
Core networks of DAT, SERT, and NET with a proteome-informed ML workflow for anticocaine addiction drug discovery. The figure highlights an autoencoder-based ML approach for encoding inhibitors/antagonists of proteins in DAT, SERT, and NET networks, predicting binding affinities, and identifying drug leads. Key processes include screening DAT, SERT, or NET inhibitor datasets and repurposing inhibitors/antagonists from other targets, followed by ADMET screening to refine potential leads. Reproduced with permission from ref. 14. Copyright 2022 ACS Publications.

Furthermore, Feng *et al.*^14^ applied machine learning and deep learning^78,79^ to address cocaine addiction, focusing on DAT, SERT, and norepinephrine transporters (NET), as shown in Fig. 4. Their study analyzed PPI networks of 61 protein targets, screening 115 407 inhibitors for repurposing potential and side effects using autoencoders, gradient-boosted decision trees (GBDT), and multitask deep neural networks (DNNs). Another notable advancement is the proteome-informed machine learning platform introduced by Gao *et al.*,^61^ which analyzes protein–protein interaction networks related to cocaine dependence and screens more than 60 000 drug candidates. This platform evaluates side effects, repurposing potential, and ADMET properties, ultimately identifying several promising lead compounds despite the failure of many existing drugs in the screenings.

**Fig. 5:**
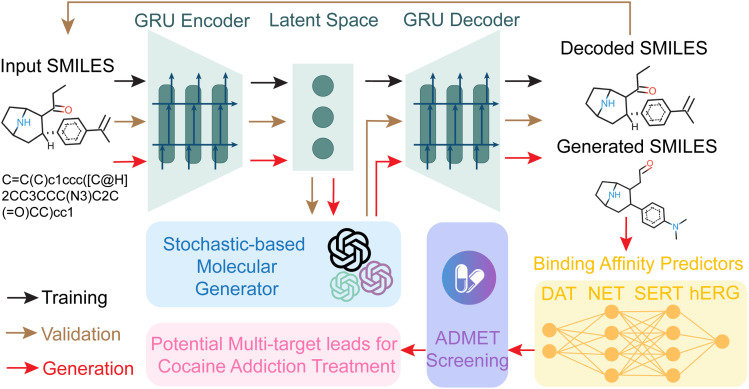
Overview of the stochastic-based generative network complex (SGNC) workflow. The workflow is depicted with distinct arrow colors: dark arrows represent the training process, brown arrows indicate validation, and red arrows denote generation. The SGNC framework includes four main components: (1) a sequence-to-sequence AutoEncoder (green) for encoding and decoding molecular data, (2) binding affinity predictors (yellow) for assessing interactions, (3) a stochastic-based molecular generator (blue) for creating novel molecules, and (4) ADMET screening *via* ADMETlab 2.0 (purple) to evaluate absorption, distribution, metabolism, excretion, and toxicity properties. Reproduced with permission from ref. 65. Copyright 2023 ACS Publications.

Kim *et al.*^64^ employed generative adversarial networks (GANs) with weighted gene co-expression network analysis (GAN-WGCNA) to analyze gene expression data from cocaine self-administration studies, identifying Alcam and Celf4 as key regulators of addictive behavior linked to dopaminergic pathways. Taking advantage of large language models (LLMs), Wang *et al.*^65^ highlighted the innovative application of ChatGPT as a virtual guide for anti-cocaine drug discovery, as shown in Fig. 5. Guided by GPT-4, a stochastic approach was integrated into the GNC model to optimize the latent space for multi-target lead generation targeting DAT, NET, and SERT using the Langevin equation. This approach integrates autoencoders, ADMET screening, and multi-target optimization, facilitating the collaboration of AI and humans to generate optimized drug candidates. These studies collectively illustrate the power of AI in uncovering dopaminergic targets and accelerating the development of anti-addiction therapies, particularly for stimulant-related disorders.

#### AI approaches to the GABAergic system

The GABAergic system, involved in inhibitory control and sedation, plays a significant role in addiction modulation and has also been explored using AI-driven methods. Goodman-Meza *et al.*^73^ applied text mining techniques and ML to classify substances associated with overdose deaths, including those affecting the GABAergic system (*e.g.*, benzodiazepines), from 35 433 medical records, and the workflow is shown in Fig. 2. This work achieved excellent classification performance, supporting target identification and hypothesis generation through large-scale literature mining and indirectly aiding GABAergic-related research. Waters *et al.*^80^ utilized QSAR models to evaluate GABA-A receptor binding of emerging benzodiazepines, predicting their addiction and abuse potential. This approach provided insights into how structural modifications influence receptor interactions, contributing to the assessment of GABAergic compounds in addiction contexts. Additionally, Jiang *et al.*^81^ introduced a proteomic learning approach to develop anesthetic agents targeting 24 GABA receptor subtypes, including GABRA5. Using PPI and DTI networks, ML screens and optimizes compounds to enhance GABA receptor inhibition while minimizing side effects. The study highlights how allosteric modulation of GABA-A receptors, sensitive to benzodiazepines and general anesthetics, disrupts GABAergic timing to induce anesthesia. This mechanism also applies to addiction therapy, as modulating GABA-A receptors can regulate neural excitability and reward pathways. By repurposing or designing high-affinity compounds like chloramphenicol and darolutamide, this approach offers a dual-purpose strategy for both anesthesia and addiction treatment.

Beyond these examples, general methodologies like reverse virtual screening and generative models (*e.g.*, VAEs and GANs) could be adapted to identify GABAergic targets. For instance, the reverse virtual screening strategy developed by Schottlender *et al.*^82^ was originally designed for antimicrobial targets. However, this strategy could also be used for screening protein libraries to predict interactions with GABA receptors, supporting drug repurposing efforts. Similarly, generative models like those used for cocaine studies^64^ could analyze GABAergic gene expression or metabolomics data to uncover novel targets. These approaches, while not directly applied to the GABAergic system, suggest potential extensions of AI-driven discovery, enhancing the identification and optimization of compounds modulating inhibitory pathways in addiction treatment.

#### Reducing hERG affinities for anti-addiction inhibitors

In the development of anti-addiction inhibitors as medications, it is necessary to avoid off-target binding that can produce unwanted side effects or toxicities. The blockade of a potassium channel, *i.e.*, human Ether-à-go-go, is a major side effect that can lead to potentially lethal ventricular tachycardia. Lee *et al.*^45^ study the reduction of hERG affinities for DAT inhibitors by combining machine learning and molecular modeling. A similar approach was also used by Fant *et al.*^46^ Various other machine learning approaches have been developed for hERG binding, such as a machine learning method proposed by Zhang *et al.*^44^ for accurate classification of hERG blockers/nonblockers. Additionally, a topological-Laplacian-assisted AI model was developed by Feng and Wei^70^ for virtual screening of the DrugBank database for hERG blockers, and a deep learning approach was introduced by Chen *et al.*^83^ for the prediction of hERG blockers.

## 4 Challenges and opportunities

### 4.1 Challenges and limitations

AI-driven drug discovery for addiction faces significant challenges in the technical, biological, and ethical domains. One of the main hurdles is the scarcity of labeled data specific to addiction-related drug targets, which AI models, particularly deep learning approaches, require to train effectively.^84,85^ This data shortage, combined with scalability issues, limits the application of traditional machine learning tools in handling the large datasets typical in drug discovery.^15^

The complicated nature of addiction systems creates additional challenges in the process. The complex interactions between multiple neurotransmitter pathways including dopamine, opioid, and glutamate contribute to the challenge of accurately predicting drug efficacy using AI models.^65^ Emerging research also points to underexplored systems—such as noradrenergic, cholinergic, histaminergic, and neuropeptide pathways (*e.g.*, orexin, CRF)—which remain poorly integrated into AI frameworks due to insufficient biological data and unclear biomarkers for treatment outcomes.^15^ This lack of biomarkers hinders validation, particularly for novel targets beyond the well-studied opioid and dopaminergic systems.

Ethical concerns are also significant, particularly concerning the interpretability of AI models. Their “black box” nature makes it challenging to trace decision-making processes, raising issues of transparency.^86^ Topological deep learning, first introduced by Cang and Wei in 2017,^87^ offers better interpretability in terms of topological invariants. Additionally, biases in training data can skew predictions, underscoring the need for fairness and representativeness.^84^ There are many approaches for imbalanced data in molecular science.^88^ Moreover, small data is another challenge in drug discovery.^85^ Data privacy poses another ethical challenge, as sensitive patient records used in addiction studies require stringent protection, yet centralized data sharing is often impractical.

The swift development of AI technology has resulted in a regulatory gray area that requires agencies like the FDA to develop suitable regulatory frameworks.^89^ However, AI algorithm opacity creates difficulties for decision traceability in clinical trials.^89^ Assessment procedures require updates to include AI prediction validation using historical data analysis. Although AI technology speeds up drug discovery processes its effectiveness must be confirmed through rigorous experimental validation to ensure applicability outside of laboratory conditions.^90^ Drug candidate verification requires an iterative feedback loop connecting AI predictions with experimental data to establish safety and efficacy. Solving regulatory and validation issues remains essential for AI to become effective in addiction treatment.

### 4.2 Trends and directions

Despite these challenges, recent advances in AI and related technologies are driving transformative progress in addiction-related drug discovery. AI-powered structure-based drug discovery is enabling researchers to analyze molecular interactions with unprecedented precision by combining machine learning with physics-based simulations.^91^ This integration facilitates the design of highly targeted and effective therapeutic compounds.

AI-driven virtual screening represents a highly promising avenue in drug discovery.^92^ By leveraging advanced NLP models to generate embeddings for both targets and drugs, this approach eliminates the need for costly molecular docking procedures. Complementing this, molecular docking methods, a cornerstone of lead compound identification in general drug discovery, systematically predict ligand binding poses to protein targets using scoring functions (*e.g.*, force-field, empirical, or knowledge-based approaches).^93^ These methods, transferable to addiction research, can accelerate the discovery of leads targeting receptors like DAT or mu-opioid receptors when enhanced by AI-driven scoring improvements. Moreover, AI enables automated, large-scale virtual screening across multiple databases, significantly enhancing efficiency and scalability.

To overcome the challenge of data scarcity, transfer learning or multitask learning has emerged as a valuable approach.^87^ This approach can be further enhanced with boosting tree-assisted multitask deep learning for small scientific datasets.^94^ These techniques adapt insights from broader datasets to addiction research, enhancing model accuracy for data-limited systems like GABAergic pathways. Federated learning also addresses data limitations by enabling collaborative model development without requiring organizations to share sensitive data, ensuring privacy and ethical compliance.^95,96^

Generative AI methods, widely used in drug discovery for lead generation, such as variational autoencoders (VAEs), generative adversarial networks (GANs), and diffusion models, are revolutionizing compound design.^85,97^ In addiction research, these approaches have generated novel compounds for cocaine addiction (*e.g.*, targeting Alcam and Celf4),^64^ while diffusion models could optimize molecules for multiple addiction-related targets like μ-opioid receptors by balancing efficacy and safety profiles,^63^ demonstrating their adaptability to anti-addiction efforts. These widely applicable generative approaches provide a powerful tool to overcome data limitations in anti-addiction efforts, mirroring their success in broader drug discovery contexts.

Another promising trend is the integration of AI with high-throughput experimental techniques, such as cryo-electron microscopy (cryo-EM)^98^ and high-content screening.^99^ This synergy accelerates the discovery and validation of drug candidates, significantly shortening the path to clinical application. In addition, AI-driven precision medicine approaches are revolutionizing addiction treatment by tailoring therapies to individual patient characteristics, such as genetic profiles and medication responses. This personalized approach improves treatment efficacy and reduces relapse rates.^84^ For lead optimization, general-purpose methods like quantitative structure–activity relationship (QSAR) modeling and multi-objective optimization, prevalent in drug discovery, are highly transferable.^100^ QSAR, enhanced by deep learning (*e.g.*, transformer models), predicts binding affinity and abuse potential for compounds targeting receptors like GABA-A or CB1,^80,101^ while multi-objective optimization balances potency, selectivity, and ADMET properties, as applied to opioid receptor–ligands,^63^ offering robust tools for refining anti-addiction candidates. Furthermore, the integration of (multi-)omics data and advanced data analysis techniques is rapidly emerging as a transformative approach to anti-addiction drug discovery.^67^ In particular, spatial transcriptomic analysis facilitates precise target identification and provides an efficient means to evaluate the effectiveness of anti-addiction drugs.

Expanding beyond current targets, underexplored biological systems present significant opportunities.^67^ The noradrenergic system, tied to stress and withdrawal, and neuropeptide systems like orexin (craving) and corticotropin-releasing factor (CRF, stress responses) remain underutilized in AI-driven addiction research. Integrating these into existing frameworks, such as topological data analysis or knowledge graphs, could yield holistic treatments, though challenges include generating sufficient biological data and defining clear therapeutic endpoints.

Advanced computational methods also offer untapped potential. AlphaFold's protein structure prediction could resolve 3D structures of addiction-related proteins in less-studied systems, enhancing virtual screening precision.^102^ Quantum computing, though computationally intensive, promises to simulate complex molecular interactions with unmatched accuracy, potentially revolutionizing multi-target drug design for addiction. Overcoming barriers like cost and expertise will be key to their adoption.

The application of LLMs in anti-addiction drug discovery is expected to become a prominent topic of interest.^65^ Using their capacity to analyze vast datasets, handle complex information, and uncover insights that were previously challenging to obtain, LLMs hold significant promise for transforming the drug discovery process.

Mathematical deep learning (MathDL) and topological deep learning (TDL) have achieved remarkable success in drug design.^87^ These methodologies emerged as top performers in the D3R Grand Challenges, a global competition series focused on advancing computer-aided drug design.^103,104^ The continued development of mathematical AI promises to drive innovation and create transformative methods in drug discovery.

Together, these advancements highlight AI's potential to overcome existing barriers and provide innovative solutions for addiction treatment. Continued interdisciplinary collaboration among AI experts, addiction researchers, and healthcare professionals will be crucial to address the multifaceted challenges of this field and advance the development of effective patient-centric therapies.

## Author contributions

Chen collected references and drafted the initial manuscript. Jiang, Hayes, and Su revised the manuscript. Wei supervised the project, obtained funding, and revised the manuscript. All authors approved the final version of the manuscript.

## Conflicts of interest

The authors declare that they have no competing interests.

## Data Availability

This review does not include any new data. All data referenced are available in the cited sources.
